# Calculation of displacements and internal forces of anchored retaining piles

**DOI:** 10.1371/journal.pone.0243659

**Published:** 2020-12-10

**Authors:** Xiaoyi Yuan, Longzhu Chen, Jianliang Deng

**Affiliations:** School of Naval Architechture, Ocean & Civil Engineering, Shanghai Jiao Tong University, Shanghai, China; China University of Mining and Technology, CHINA

## Abstract

Pile-anchor retaining structures are widely used in excavation engineering. The evaluation of lateral displacements, the internal forces of piles are extremely important for the performance of the structure. Most of the existing methods are empirical, semiempirical or FEM methods, while analytic calculation methods for this evaluation are rare. This paper presents an analytic method to calculate the displacements and internal forces of anchored retaining piles based on the existing design code. In the calculation method, the singular function is applied to evaluate the effect of segmented loading on the deflection of a beam with a nonuniform cross section. The load concentration function, expressed by the singular function, can describe the segmented load and be integrated without a complicated procedure for determining the integral constants. The method is applied to a structure in Wenzhou, China, and the calculation results are compared to the field measurement results. This method is only valid for pre-failure predictions.

## Introduction

Pile-anchor retaining structures, characterized by reliable sustainability, limited space occupation, convenient construction and low cost, have been widely used in deep excavation engineering in recent years. Controlling the deformation and internal forces of retaining piles is an essential issue in the design of pile-anchor retaining structures. Most of the existing calculation methods for the control of the deformation are empirical, semi-empirical or FEM methods. In addition, the existing design code also provides some effective calculation methods.

Empirical and semi-empirical studies, based on a large amount of engineering practices, have been carried out. Based on analyses of more than 530 case studies on deep excavations, the empirical analysis indicated that the maximum lateral displacement and ground movements always take place in a certain section of the wall, and the retaining wall and ground movements seem to be determined by other relevant factors while the system has sufficient stiffness [1]. A semi-empirical formula was proposed based on the finite element analysis of 1032 hypothetical cases, wherein the polynomial regression method was used to calculate the maximum lateral displacement of the retaining piles [2]. However, the assumptions of the averaged soil properties and continuous external forces largely decrease the precision of both the empirical and semi-empirical analysis results. In realistic engineering cases, piles in excavation engineering usually pass through several soil layers with different soil mechanics characteristics and are retained by different types of structures that cause loads that act on the retaining piles; several types of external forces or pressure acting on the retaining piles are often discontinuous, such as the anchor force, earth pressure and groundwater pressure.

Numerical methods have been widely used in the design of retaining structures in excavation engineering. Based on the results from 30 nonlinear finite element analyses of undrained deep excavations in stiff clay, the displacement flexibility number method was proposed. This theory indicates that retaining structures with the same displacement flexibility number tend to have contiguous maximum lateral displacements, and this conclusion is the same as that drawn by Addenbrooke in 1994 [3]. In addition, these analyses have further demonstrated that when the stiffness of a retaining structure is beyond a certain threshold, it only slightly affects the displacement [4]. Elastic-plastic analysis using numerical methods was carried out to calculate the internal forces and displacement of a pile-anchor retaining structure during the process of excavation [5]. A finite element program PLAXIS was introduced to locate the position of the potential failure plane and safety factor of the excavations. Furthermore, it analyzed the influencing factors of the stability of the retaining structures and excavations [6]. The excavation of Sukhumvit Station was studied using the FEM and empirical methods to investigate the D-wall movements and ground surface settlements [7]. However, the predictions of the numerical methods depend largely on the modelling of the constitutive relationship of the soil and the selection of parameters by trial and error.

Meanwhile, several theoretical methods have been proposed for the calculations of pile-anchor retaining structures. A simplified method was proposed by means of quasi-elastic summation to calculate the lateral displacement versus depth; however, this method is only suitable for braced excavations with small displacements at the bottom of the retaining wall [8]. An analytical solution for laterally loaded piles in soils with a stiffness that linearly increases with depth was proposed by introducing a Fourier-Laplace integral, and a simplified expression for this solution can be used to calculate the deflection and bending movement profiles of the piles with high accuracy and a small amount of calculation effort [9].

The design code *(JGJ, 2012)* summarizes some calculation methods for the deformation of pile-anchor retaining structures [10]. The design code *(JGJ, 2012)* shows that the *m-method* is an effective method for determining the soil pressure under the pit bottom of a pile-anchor retaining structure. This method assumes that the soil under the bottom of the excavation is elastic and that the Young’s modulus of the soil increases linearly with depth. Further research proved that the *m-method* and its improved version are effective in engineering practice. However, the whole calculation in the code may be too conservative, inducing an unprecise prediction of the deformation of the pile-anchor retaining structure.

By introducing the *singular function method*, this paper presents an analytical method characterized by simple computation and reliable results to calculate the pre-failure displacements and internal forces of anchored retaining piles.

## Calculation of internal forces

**Fig 1:**
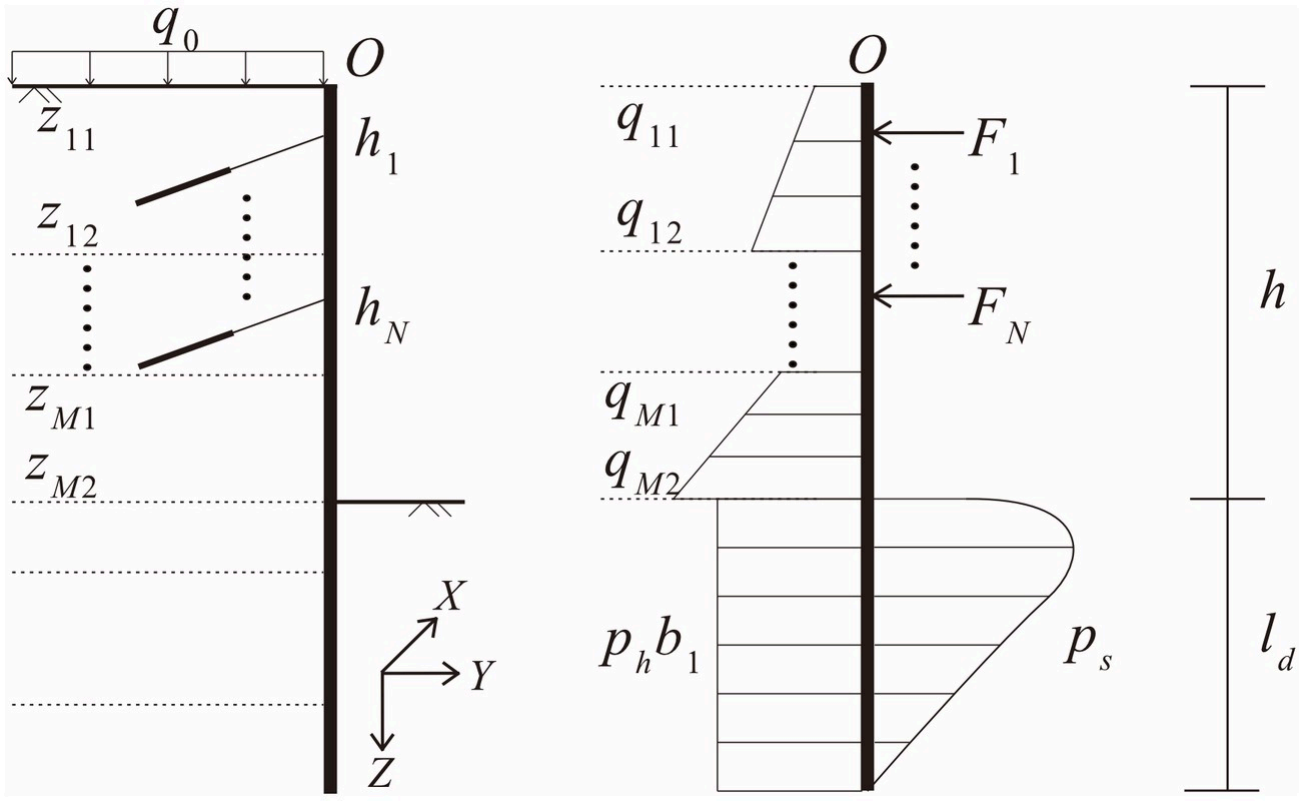
Calculation model of a pile-anchored retaining structure.

A typical pile-anchor retaining structure and discontinuous horizontal loads are shown in Fig 1. There are *M* layers of soil and *N* composite anchors above the pit bottom in this model. The assumptions in the calculation are defined as follows: (1) the calculation is a plane strain problem, and the calculated width of the retaining pile along the x-axis is *b*_1_; (2) the retaining pile is a linear elastic vertical beam with a constant cross section size without a moment or shear force acting at its top end; (3) Rankine’s earth pressure theory is applicable for the soil above the pit bottom, while the *m-method* is applicable for the soil below the pit bottom; (4) the effects of the pile’s vertical displacements are ignored; and (5) the soil under the pit bottom is homogeneous. This method is intended to give pre-failure predictions, and shall not be used for post-failure situations.

### Equilibrium differential equations of pile

The analytical solution for the displacements and internal forces for the structure shown in Fig 1 is usually based on a segmented function. The retaining pile must be segmented at each joint of the anchors and each interface of the soil layers, in which case the equilibrium differential equations would be composed of (*M* + *N* + 1) equations and there would be 4(*M* + *N* + 1) integral constants to be determined. Furthermore, a universal solution could not be given since each specific engineering case has different values for *M* and *N*, and the equations need to be segmented in different ways.

The singular function can express a discontinuous (segmented) function in a continuous form and has a calculus algorithm that is essentially the same as a continuous function. Therefore, the *singular function method* is used in the calculation to accurately evaluate the effects of the discontinuous loads on the deformation of retaining piles [11].

The discontinuous loads acting on the retaining pile can be expressed in a single expression that has a continuous form using the *singular function method* as shown in Eq 1:

$$\begin{array}{c} q\left(z\right)=\sum_{i=1}^{M}q_{i}\left(z\right)+\sum_{j=1}^{N}p_{j}\left(z\right)\; \end{array}$$ (1)

where *p*_*j*_(*z*) = *k*_*j*_ *y*_*j*_〈*z* − *h*_*j*_〉^−1^ is the resistance of the *j*^th^ anchor acting on the retaining pile, and $q_{i}\left(z\right)=b_{1}[q_{i1}{\langle z-z_{i1}\rangle }^{0}-q_{i2}{\langle z-z_{i2}\rangle }^{0}+\frac{q_{i2}-q_{i1}}{z_{i2}-z_{i1}}({\langle z-z_{i1}\rangle }^{1}-{\langle z-z_{i2}\rangle }^{1})]$ is the earth pressure of the *i*^th^ soil layer, which is calculated using Rankine’s earth pressure theory. Here *k*_*j*_ is tensile stiffness of the anchor and *b*_1_ is the calculative width of the pile.

The equilibrium differential equation of the pile above the pit bottom is thus shown in Eq 2:

$$\begin{array}{c} EI\frac{d^{4}y}{dz^{4}}=q\left(z\right) \end{array}$$ (2)

where *EI* is the flexural rigidity of the retaining pile.

The equilibrium differential equation below the pit bottom is shown in Eq 3:

$$\begin{array}{c} EI\frac{d^{4}y}{dz^{4}}=-Kb_{1}y+p_{h}b_{1} \end{array}$$ (3)

where *K* is a coefficient related to the soil layers below the pit bottom and *p*_*h*_ is the horizontal active pressure due to the soil layers above the pit bottom.

The expressions for *K* and *p*_*h*_ are given by Eqs 4 and 5, respectively.

$$\begin{array}{c} K=m{\left(z-h\right)}^{\frac{1}{n}}+m_{0} \end{array}$$ (4)

where *n* is a positive integer, *m* and *m*_0_ are coefficients.

$$\begin{array}{c} p_{h}=(\sum \gamma_{i}h_{i}+q_{0}){\bar{K}}_{a}-2\bar{c}\sqrt{{\bar{K}}_{a}} \end{array}$$ (5)

where ${\bar{K}}_{a}=\tan^{2}(45^{{}^{\circ}}-\bar{\varphi }/2)$; ∑*γ*_*i*_ *h*_*i*_ is the sum of the dead weight of the soil layers above the pit bottom; *q*_0_ is the ground overload; and $\bar{c}$ and $\bar{\varphi }$ are the average cohesion and internal friction angle weighted by the thickness of the soil layers above the pit bottom, respectively.

**Fig 2:**
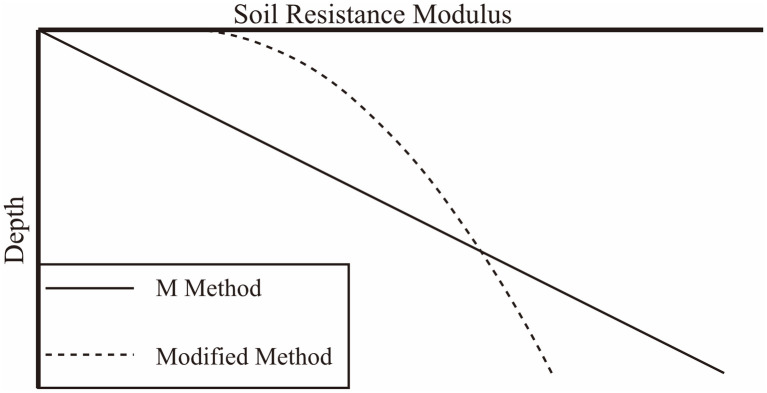
Soil resistance module for the *m-method* and the modified method.

It is suggested in the *m-method* that *K* = *m*(*z* − *h*), indicating that the module of soil resistance at the pit bottom is zero and increases linearly with depth, as shown in Fig 2. Obviously, the modified formula can be not only linear, and the soil resistance at the pit bottom can also be non-zero. Thus, the formula could be closer to practical engineering.

### Solution of differential equations

#### Solution of Eq 2

Eq 2 is a 4^th^ order ordinary differential equation that can be solved by direct integration using the *singular function method*. Considering that the top end of the pile bears no shear force or moment, the solution is shown in Eq 6:

$$\begin{array}{c} \begin{array}{cc} EIy & =\sum_{i=1}^{M}[\frac{q_{i1}}{24}{\left\langle z-z_{i1}\right\rangle }^{4}-\frac{q_{i2}}{24}{\left\langle z-z_{i2}\right\rangle }^{4}+\frac{q_{i2}-q_{i1}}{120(z_{i2}-z_{i1})}({\left\langle z-z_{i1}\right\rangle }^{5}-{\left\langle z-z_{i2}\right\rangle }^{5})]\\ & -\sum_{j=1}^{N}\frac{1}{6}k_{j}y_{j}{\left\langle z-h_{j}\right\rangle }^{3}+C_{3}z+C_{4} \end{array} \end{array}$$ (6)

where *C*_3_ and *C*_4_ are integral constants.

According to the mechanics of materials, the rotation of the pile is:

$$\begin{array}{c} \begin{array}{cc} EI\varphi & =\sum_{i=1}^{M}[\frac{q_{i1}}{6}{\left\langle z-z_{i1}\right\rangle }^{3}-\frac{q_{i2}}{6}{\left\langle z-z_{i2}\right\rangle }^{3}+\frac{q_{i2}-q_{i1}}{24(z_{i2}-z_{i1})}({\left\langle z-z_{i1}\right\rangle }^{4}-{\left\langle z-z_{i2}\right\rangle }^{4})]\\ & -\sum_{j=1}^{N}\frac{1}{2}k_{j}y_{j}{\left\langle z-h_{j}\right\rangle }^{2}+C_{3} \end{array} \end{array}$$ (7)

Assume that when *z* = *h*, the rotation and displacement of the pile are *φ*_*b*−_ and *y*_*b*−_, respectively. The symbol “-” in the subscript here means that the displacement and rotation of the pile are calculated from the equilibrium differential equation above the pit bottom.

#### Solution of Eq 3

Eq 3 is a 4^th^ order non-homogeneous differential equation with variable coefficients. A series method was introduced to solve the equation. Here, the derivation is given when *m*_0_ = 0, *n* ∈ *Z*.

Let $\alpha ={\left(\frac{mb_{1}}{EI}\right)}^{\frac{1}{4+\frac{1}{n}}}$ and *x* = *z* − *h*, then we have:

$$\begin{array}{c} \frac{d^{4}y}{dx^{4}}=-\alpha^{4+\frac{1}{n}}x^{\frac{1}{n}}y+\frac{p_{h}b_{1}}{EI} \end{array}$$ (8)

Assume that *x* = *t*^*n*^, namely, $t=x^{\frac{1}{n}}$, then:

$$\begin{array}{c} \begin{array}{cc} \frac{d^{4}y}{dx^{4}} & =\frac{1}{n^{4}}[-(n-1)(2n-1)(3n-1)t^{-4n+1}\frac{dy}{dt}+(11n-7)(n-1)t^{-4n+2}\frac{d^{2}y}{dt^{2}}-6(n-1)t^{-4n+3}\frac{d^{3}y}{dt^{3}}+t^{-4n+4}\frac{d^{4}y}{dt^{4}}] \end{array} \end{array}$$ (9)

Using the series method, assume that the solution of Eq 3 is $y=\sum_{r=0}^{\infty }a_{r}t^{r}$, then:

$$\begin{array}{c} t^{-4n+1}\frac{dy}{dt}=\sum_{r=0}^{\infty }ra_{r}t^{r-4n} \end{array}$$ (10)

$$\begin{array}{c} t^{-4n+2}\frac{d^{2}y}{dt^{2}}=\sum_{r=0}^{\infty }r(r-1)a_{r}t^{r-4n} \end{array}$$ (11)

$$\begin{array}{c} t^{-4n+3}\frac{d^{3}y}{dt^{3}}=\sum_{r=0}^{\infty }r(r-1)(r-2)a_{r}t^{r-4n} \end{array}$$ (12)

$$\begin{array}{c} t^{-4n+4}\frac{d^{4}y}{dt^{4}}=\sum_{r=0}^{\infty }r(r-1)(r-2)(r-3)a_{r}t^{r-4n} \end{array}$$ (13)

By substituting Eqs 10–13 into Eq 9, we obtain:

$$\begin{array}{c} \sum_{r=0}^{\infty }r(r-n)(r-2n)(r-3n)a_{r}t^{r-4n}=-n^{4}\alpha^{4+\frac{1}{n}}\sum_{r=0}^{\infty }a_{r}t^{r+1}+\frac{n^{4}p_{h}b_{1}}{EI} \end{array}$$ (14)

Note that Eq 14 shall be tenable for every value of *t*, thus:

1. *r* < 4*n*, there is *r*(*r* − *n*)(*r* − 2*n*)(*r* − 3*n*)*a*_*r*_ = 0.
  Thus, *a*_*r*_ can be none-zero if and only if *r* = 0, *n*, 2*n*, 3*n*;
2. *r* = 4*n*, there is $4n(4n-n)(4n-2n)(4n-3n)a_{4n}=\frac{n^{4}p_{h}b_{1}}{EI}$.
  Thus, $a_{4n}=\frac{p_{h}b_{1}}{24EI}$;
3. *r* > 4*n*, there is $r(r-n)(r-2n)(r-3n)a_{r}=-n^{4}\alpha^{4+\frac{1}{n}}a_{r-(4n+1)}$.
  Thus, $a_{r}=-\frac{n^{4}\alpha^{4+\frac{1}{n}}}{r(r-n)(r-2n)(r-3n)}a_{r-(4n+1)}$.

Let *r* = (4*n* + 1)*s* + *R*, *R* = 0, *n*, 2*n*, 3*n*, 4*n*, then the general term formula of *a*_*r*_ is:

$$\begin{array}{c} a_{(4n+1)s+R}=\frac{{\left(-1\right)}^{s}{\left(n^{4}\alpha^{4+\frac{1}{n}}\right)}^{s}}{{\left\{s\right\}}_{R}^{!!}}a_{R} \end{array}$$ (15)

where ${{\left\{s\right\}}^{!!}}_{R}=\prod_{k=1}^{s}\prod_{i=0}^{3}[(4n+1)k+R-in]$, *s* = 1, 2, ⋯, ∞. When *r* ≠ (4*n* + 1)*s* + *R*, *a*_*r*_ = 0.

Thus, the solution of Eq 3 is:

$$\begin{array}{c} \begin{array}{cc} y & =a_{0}[1+\sum_{s=1}^{\infty }\frac{{\left(-1\right)}^{s}n^{4s}}{{\left\{s\right\}}_{R=0}^{!!}}{\left(\alpha x\right)}^{\frac{4n+1}{n}s}]\\ & +a_{n}[x+\sum_{s=1}^{\infty }\frac{{\left(-1\right)}^{s}n^{4s}}{{\left\{s\right\}}_{R=n}^{!!}}\frac{1}{\alpha }{\left(\alpha x\right)}^{\frac{4n+1}{n}s+1}]\\ & +a_{2n}[x^{2}+\sum_{s=1}^{\infty }\frac{{\left(-1\right)}^{s}n^{4s}}{{\left\{s\right\}}_{R=2n}^{!!}}\frac{1}{\alpha^{2}}{\left(\alpha x\right)}^{\frac{4n+1}{n}s+2}]\\ & +a_{3n}[x^{3}+\sum_{s=1}^{\infty }\frac{{\left(-1\right)}^{s}n^{4s}}{{\left\{s\right\}}_{R=3n}^{!!}}\frac{1}{\alpha^{3}}{\left(\alpha x\right)}^{\frac{4n+1}{n}s+3}]\\ & +a_{4n}[x^{4}+\sum_{s=1}^{\infty }\frac{{\left(-1\right)}^{s}n^{4s}}{{\left\{s\right\}}_{R=4n}^{!!}}\frac{1}{\alpha^{4}}{\left(\alpha x\right)}^{\frac{4n+1}{n}s+4}] \end{array} \end{array}$$ (16)

This series can be proven mathematically to be absolutely convergent.

Assuming that the deflection, rotation, moment and shear force of the retaining pile at the bottom of the excavation are *y*_*b*_, *φ*_*b*_, *M*_*b*_ and *Q*_*b*_, respectively, then *y*|_*x*=0_ = *a*_0_ = *y*_*b*_, *y*′|_*x*=0_ = *a*_*n*_ = *φ*_*b*_, $y^{\prime \prime }{|}_{x=0}=2a_{2n}=\frac{M_{b}}{EI}$ and $y^{\prime \prime \prime }{|}_{x=0}=6a_{3n}=\frac{Q_{b}}{EI}.$ Then, Eq 16 can be simplified as follows:

$$\begin{array}{c} \begin{array}{c} y=y_{b}A(\alpha x)+\frac{\varphi_{b}}{\alpha }B(\alpha x)+\frac{M_{b}}{\alpha^{2}EI}C(\alpha x)+\frac{Q_{b}}{\alpha^{3}EI}D(\alpha x)+\frac{p_{h}b_{1}}{\alpha^{4}EI}E(\alpha x) \end{array} \end{array}$$ (17)

where $A(\alpha x)=1+\sum_{s=1}^{\infty }\frac{{\left(-1\right)}^{s}n^{4s}}{{\left\{s\right\}}_{R=0}^{!!}}{\left(\alpha x\right)}^{\frac{4n+1}{n}s}$, $B(\alpha x)=\alpha x+\sum_{s=1}^{\infty }\frac{{\left(-1\right)}^{s}n^{4s}}{{\left\{s\right\}}_{R=n}^{!!}}{\left(\alpha x\right)}^{\frac{4n+1}{n}s+1}$, $C(\alpha x)=\frac{{\left(\alpha x\right)}^{2}}{2}+\sum_{s=1}^{\infty }\frac{{\left(-1\right)}^{s}n^{4s}}{2{\left\{s\right\}}_{R=2n}^{!!}}{\left(\alpha x\right)}^{\frac{4n+1}{n}s+2}$, $D(\alpha x)=\frac{{\left(\alpha x\right)}^{3}}{6}+\sum_{s=1}^{\infty }\frac{{\left(-1\right)}^{s}n^{4s}}{6{\left\{s\right\}}_{R=3n}^{!!}}{\left(\alpha x\right)}^{\frac{4n+1}{n}s+3}$, $E(\alpha x)=\frac{{\left(\alpha x\right)}^{4}}{24}+\sum_{s=1}^{\infty }\frac{{\left(-1\right)}^{s}n^{4s}}{24{\left\{s\right\}}_{R=4n}^{!!}}{\left(\alpha x\right)}^{\frac{4n+1}{n}s+4}.$

In addition, the rotation of the pile is:

$$\begin{array}{c} \frac{\varphi }{\alpha }=y_{b}A^{\prime }(\alpha x)+\frac{\varphi_{b}}{\alpha }B^{\prime }(\alpha x)+\frac{M_{b}}{\alpha^{2}EI}C^{\prime }(\alpha x)+\frac{Q_{b}}{\alpha^{3}EI}D^{\prime }(\alpha x)+\frac{p_{h}b_{1}}{\alpha^{4}EI}E^{\prime }(\alpha x) \end{array}$$ (18)

where $A^{\prime }(\alpha x)=\frac{d}{d(\alpha x)}A(\alpha x)$, $B^{\prime }(\alpha x)=\frac{d}{d(\alpha x)}B(\alpha x)$ and so on.

Assume that when *x* = 0, the rotation and displacement of the pile are *φ*_*b*+_ and *y*_*b*+_, respectively. The symbol “+” in the subscript here means that the displacement and rotation of the pile are calculated from the equilibrium differential equation below the pit bottom.

When *m*_0_ ≠ 0, it can be proved in similar way that the expressions of the solution have the same form as Eqs 17 and 18, except that the expressions of *A*(*αx*) ∼ *E*(*αx*) cannot be given explicitly.

#### Calculation of unknown parameters

There are a total of 6 unknown parameters (*C*_3_, *C*_4_, *Q*_*b*_, *M*_*b*_, *y*_*b*_ and *φ*_*b*_) that can be calculated using boundary conditions and continuity conditions.

Through static equilibrium analysis of the pile above the pit bottom, the shear force (*Q*_*b*_) and moment (*M*_*b*_) of the pile at the pit bottom are:

$$\begin{array}{c} \begin{array}{cc} Q_{b} & =\int_{0}^{h}q(z)dz\\ & =\frac{1}{2}\sum_{i=1}^{M}b_{1}(q_{i1}+q_{i2})(z_{i2}-z_{i1})-\sum_{j=1}^{N}k_{j}y_{j} \end{array} \end{array}$$ (19)

and

$$\begin{array}{c} \begin{array}{cc} M_{b} & =\int_{0}^{h}q(z)(h-z)dz\\ & =\frac{1}{6}\sum_{i=1}^{M}b_{1}(z_{i2}-z_{iz})[q_{i1}(3h-z_{i2}-2z_{i1})+q_{i2}(3h-2z_{i2}-z_{i1})]\\ & -\sum_{j=1}^{N}k_{j}y_{j}(h-h_{j}) \end{array} \end{array}$$ (20)

Note that here we used the lateral displacement of anchors *y*_*j*_ for derivation, but the values of *y*_*j*_ are not known at this point. Their values will be given later.

Assume that the displacement and rotation of the pile when *x* = *l*_*d*_ is *δ* and *θ* respectively, and let *A*(*αl*_*d*_) = *A*, *A*′(*αl*_*d*_) = *A*′ and so forth. Substituting *δ* and *θ* into Eqs 17 and 18 gives the following equation:

$$\begin{array}{c} \begin{array}{cc} y_{b+} & =\frac{1}{\alpha^{4}EI(AB^{\prime }-A^{\prime }B)}[\alpha^{2}M_{b}(BC^{\prime }-B^{\prime }C)+\alpha Q_{b}(BD^{\prime }-B^{\prime }D)+p_{h}b_{1}(BE^{\prime }-B^{\prime }E)]+\frac{\alpha \delta B^{\prime }-\theta B}{\alpha (AB^{\prime }-A^{\prime }B)} \end{array} \end{array}$$ (21)

$$\begin{array}{c} \begin{array}{cc} \varphi_{b+} & =\frac{1}{\alpha^{3}EI(AB^{\prime }-A^{\prime }B)}[\alpha^{2}M_{b}(A^{\prime }C-AC^{\prime })+\alpha Q_{b}(A^{\prime }D-AD^{\prime })+p_{h}b_{1}(A^{\prime }E-AE^{\prime })]-\frac{\alpha \delta A^{\prime }-\theta A}{AB^{\prime }-A^{\prime }B} \end{array} \end{array}$$ (22)

The symbol “+” in the subscript here means that the rotation and displacement of the pile were calculated from the equilibrium differential equation below the pit bottom.

Substitute Eqs 19 and 20 into Eqs 21 and 22, and let *T*_0_ = *AB*′ − *A*′ *B*, *T*_1_ = *BC*′ − *B*′ *C*, *T*_2_ = *BD*′ − *B*′ *D*, *T*_3_ = *BE*′ − *B*′ *E*, *T*_4_ = *A*′ *C* − *AC*′, *T*_5_ = *A*′ *D* − *AD*′, *T*_6_ = *A*′ *E* − *AE*′, $P=\frac{1}{2}\sum_{i=1}^{M}b_{1}(q_{i1}+q_{i2})(z_{i2}-z_{i1})$ and $W=\frac{1}{6}\sum_{i=1}^{M}b_{1}(z_{i2}-z_{i1})[q_{i1}(3h-z_{i2}-2z_{i1})+q_{i2}(3h-2z_{i2}-z_{i1})]$; then we have:

$$\begin{array}{c} \begin{array}{cc} y_{b+} & =\frac{1}{\alpha^{4}EIT_{0}}\{\alpha^{2}WT_{1}+\alpha PT_{2}+p_{h}b_{1}T_{3}-\alpha \sum_{j=1}^{N}k_{j}[\alpha T_{1}(L-h_{j})+T_{2}]y_{j}\}\\ & +\frac{\alpha \delta B^{\prime }-\theta B}{\alpha T_{0}} \end{array} \end{array}$$ (23)

$$\begin{array}{c} \begin{array}{cc} \varphi_{b+} & =\frac{1}{\alpha^{3}EIT_{0}}\{\alpha^{2}WT_{4}+\alpha PT_{5}+p_{h}b_{1}T_{6}-\alpha \sum_{j=1}^{N}k_{j}[\alpha T_{4}(L-h_{j})+T_{5}]y_{j}\}\\ & -\frac{\alpha \delta A^{\prime }-\theta A}{T_{0}} \end{array} \end{array}$$ (24)

Considering the continuity condition of the retaining pile at the pit bottom, there are *y*_*b*−_ = *y*_*b*+_ and *φ*_*b*−_ = *φ*_*b*+_.

Let $T_{7}=\sum_{i=1}^{M}b_{1}\{\frac{q_{i1}}{6}{\left(h-z_{i1}\right)}^{3}-\frac{q_{i2}}{6}{\left(h-z_{i2}\right)}^{3}+\frac{q_{i2}-q_{i1}}{24(z_{i2}-z_{i1})}[{\left(h-z_{i1}\right)}^{4}-{\left(h-z_{i2}\right)}^{4}]\}$ and $T_{8}=\sum_{i=1}^{M}b_{1}\{\frac{q_{i1}}{24}{\left(h-z_{i1}\right)}^{4}-\frac{q_{i2}}{24}{\left(h-z_{i2}\right)}^{4}+\frac{q_{i2}-q_{i1}}{120(z_{i2}-z_{i1})}[{\left(h-z_{i1}\right)}^{5}-{\left(h-z_{i2}\right)}^{5}]\}$, then we have:

$$\begin{array}{c} \begin{array}{cc} C_{3} & =\frac{WT_{4}}{\alpha T_{0}}+\frac{PT_{5}}{\alpha^{2}T_{0}}+\frac{p_{h}b_{1}T_{6}}{\alpha^{3}T_{0}}-T_{7}-\sum_{j=1}^{N}k_{j}[\frac{\alpha T_{4}(h-h_{j})+T_{5}}{\alpha^{2}T_{0}}-\frac{{\left(h-h_{j}\right)}^{2}}{2}]y_{j}\\ & -EI\frac{\alpha \delta A^{\prime }-\theta A}{T_{0}} \end{array} \end{array}$$ (25)

$$\begin{array}{c} \begin{array}{cc} C_{4} & =\frac{W(T_{1}-\alpha T_{4}h)}{\alpha^{2}T_{0}}+\frac{P(T_{2}-\alpha T_{5}h)}{\alpha^{3}T_{0}}+\frac{p_{h}b_{1}(T_{3}-\alpha T_{6}h)}{\alpha^{4}T_{0}}-T_{8}\\ & -\sum_{j=1}^{N}k_{j}[\frac{\alpha (T_{1}-\alpha T_{4}h)(h-h_{j})}{\alpha^{3}T_{0}}+\frac{\left(T_{2}-\alpha T_{5}h\right)}{\alpha^{3}T_{0}}+\frac{{\left(h-h_{j}\right)}^{2}(2h+h_{j})}{6}]y_{j}\\ & +(T_{7}+EI\frac{\alpha \delta A^{\prime }-\theta A}{T_{0}})h+EI\frac{\alpha \delta B^{\prime }-\theta B}{\alpha T_{0}} \end{array} \end{array}$$ (26)

Now, all the unknown parameters except the values of *y*_*j*_ have been obtained. For the sake of brevity, let:

$$\begin{array}{c} \begin{array}{cc} I_{1}(z) & =\sum_{i=1}^{M}b_{1}[\frac{q_{i1}}{24}{\left\langle z-z_{i1}\right\rangle }^{4}-\frac{q_{i2}}{24}{\left\langle z-z_{i2}\right\rangle }^{4}+\frac{q_{i2}-q_{i1}}{120(z_{i2}-z_{i1})}({\left\langle z-z_{i1}\right\rangle }^{5}-{\left\langle z-z_{i2}\right\rangle }^{5})]\\ & +z(\frac{WT_{4}}{\alpha T_{0}}+\frac{PT_{5}}{\alpha^{2}T_{0}}+\frac{p_{h}b_{1}T_{6}}{\alpha^{3}T_{0}}-T_{7}-EI\frac{\alpha \delta A^{\prime }-\theta A}{T_{0}})\\ & +\frac{W(T_{1}-\alpha T_{4}h)}{\alpha^{2}T_{0}}+\frac{P(T_{2}-\alpha T_{5}h)}{\alpha^{3}T_{0}}+\frac{p_{h}b_{1}(T_{3}-\alpha T_{6}h)}{\alpha^{4}T_{0}}\\ & +(T_{7}+EI\frac{\alpha \delta A^{\prime }-\theta A}{T_{0}})h-T_{8}+EI\frac{\alpha \delta B^{\prime }-\theta B}{\alpha T_{0}} \end{array} \end{array}$$ (27)

and

$$\begin{array}{c} \begin{array}{cc} I_{2,j}(z) & =-\frac{1}{6}k_{j}{\left\langle z-h_{j}\right\rangle }^{3}-zk_{j}[\frac{\alpha T_{4}(h-h_{j})+T_{5}}{\alpha^{2}T_{0}}-\frac{{\left(h-h_{j}\right)}^{2}}{2}]\\ & -k_{j}[\frac{\alpha (T_{1}-\alpha T_{4}h)(h-h_{j})}{\alpha^{3}T_{0}}+\frac{T_{2}-\alpha T_{5}h}{\alpha^{3}T_{0}}+\frac{{\left(h-h_{j}\right)}^{2}(2h+h_{j})}{6}] \end{array} \end{array}$$ (28)

Then, Eq 6 becomes:

$$\begin{array}{c} EIy=I_{1}(z)+\sum_{j=1}^{N}I_{2,j}(z)y_{j} \end{array}$$ (29)

Note that Eq 29 shall be self-consistent, namely, for each *i* ∈ [1, *N*], there must be:

$$\begin{array}{c} EIy_{i}=I_{1}(h_{i})+\sum_{j=1}^{N}I_{2,j}(h_{i})y_{j} \end{array}$$ (30)

Also note that here $\sum_{j=1}^{N}I_{2,j}(h_{i})y_{j}$ can be rewritten in matrix form as shown in Eq 31:

$$\begin{array}{c} \sum_{j=1}^{N}I_{2,j}(h_{i})y_{j}=[I_{2,1}(h_{i}),\cdots ,I_{2,j}(h_{i})]\cdot [\begin{array}{c} y_{1}\\ y_{2}\\ \vdots \\ y_{j} \end{array}] \end{array}$$ (31)

Substituting Eq 31 into Eq 30 for each *i* ∈ [1, *N*], we got a series of equations that can be written in matrix form as shown in Eq 32:

$$\begin{array}{c} {}[U_{ij}]\vec{y}+EI\vec{y}=\vec{H} \end{array}$$ (32)

where *U*_*ij*_ = −*I*_2,*j*_(*h*_*i*_), $\vec{y}={\left[y_{1},y_{2},...y_{N}\right]}^{T}$, $\vec{H}={\left[I_{1}\left(h_{1}\right),I_{1}\left(h_{2}\right),...I_{1}\left(h_{N}\right)\right]}^{T}$.

The solution vector of Eq 32 is composed with the values of *y*_*j*_.

In addition, if the assumption of boundary conditions at the bottom of the pile is that the shear force and moment acting on the bottom of the pile are 0, it is easy to prove in similar way that the solution would still have a similar form to Eq 29 except that *T*_*n*_ (*n* = 1 ∼ 6) should be replaced by $\frac{d^{2}T_{n}}{d{\left(\alpha l_{d}\right)}^{{}^{2}}}$ (*n* = 1 ∼ 6) and the terms for *δ* and *θ* should be removed.

Using the *singular function method*, Eqs 17 and 29 can be written in one expression as Eq 33:

$$\begin{array}{c} y=Y_{1}(z){\left\langle z-h\right\rangle }^{0}+Y_{2}(z)(1-{\left\langle z-h\right\rangle }^{0}) \end{array}$$ (33)

where $Y_{1}(z)=y_{b}A[\alpha (z-h)]+\frac{\varphi_{b}}{\alpha }B[\alpha (z-h)]+\frac{M_{b}}{\alpha^{2}EI}C[\alpha (z-h)]+\frac{Q_{b}}{\alpha^{3}EI}D[\alpha (z-h)]+\frac{p_{h}b_{1}}{\alpha^{4}EI}E[\alpha (z-h)]$ and $Y_{2}(z)=\frac{1}{EI}[I_{1}(z)+\sum_{j=1}^{n}I_{2,j}(z)y_{j}]$.

Here Eq 33 is the analytic general solution of displacement of anchored retaining piles. Its derivatives are namely rotation and internal forces of the pile.

## Application of the solution

**Fig 3:**
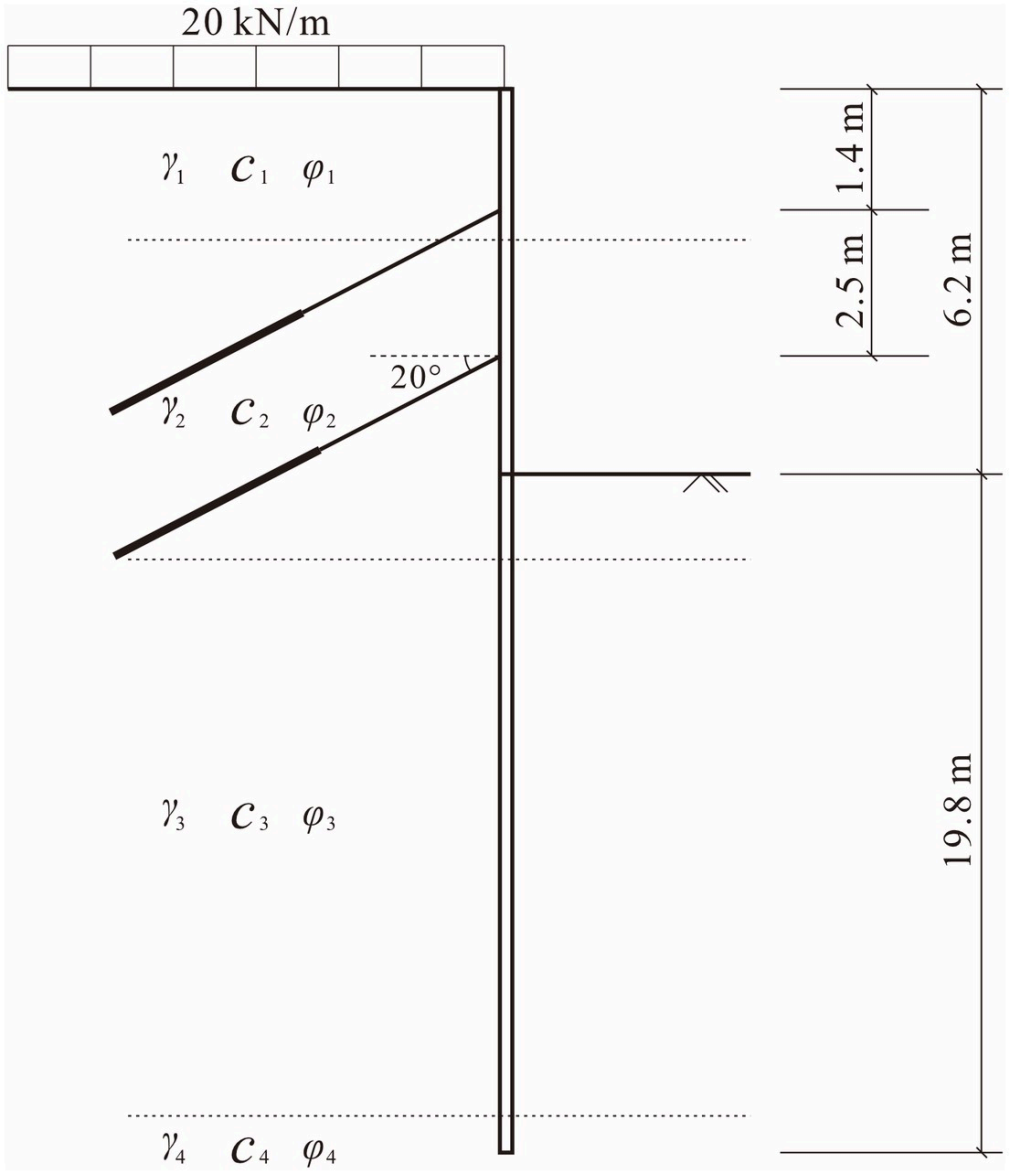
Profile of the excavation project in Wenzhou, Zhejiang, China.

The solution established above was applied to an excavation in Wenzhou, Zhejiang, China. The excavation was in thick soft clay ground consisting of mainly mud and mud silt. The excavation was 6.2 m in depth, while the pile was 26 m in length. The groundwater level was 1 m. The retaining pile had a section diameter of 0.8 m, and the center distance of adjacent the piles was 1.1 m. The piles were connected by 1.1 m × 0.8 m rectangular top beams. Two composite anchors with an inclination of 20° were placed along the retaining pile with vertical distances of 1.4 m and 3.9 m from the top of the pile, respectively, and the lateral distance between the adjacent anchors was 2.0 m. The profile of the excavation is shown in Fig 3.

The physical-mechanical parameters obtained from ground experiments and engineering experiences of the soil layers are shown in Table 1.

**Table 1:** Physical-mechanical parameters of the Wenzhou soil layer.

Sequence	Thickness (m)	Weight (kN/m^3^)	Cohesion (kPa)	Friction Angle (°)
1	2.0	17.5	14.0	12.0
2	9.0	18.0	6.0	8.5
3	14.0	17.6	7.0	8.0
4	14.8	17.1	10.0	10.5

The ground overload was 20 kPa, and the groundwater level was 1 m. Considering the soil conditions of this project, free boundary conditions were applied at both the upper and lower ends. The effect of the top beam was equivalent to an anchor with a stiffness of 1.9 × 10^3^ kN/m. Field experiments showed that the stiffness of the other anchors was 4.5 × 10^3^ kN/m which would be reduced because the calculation is assumed to be a plane strain problem. The reduction factor λ is calculated using Eq 34 according to *JGJ,2012*:

$$\begin{array}{c} \lambda =\frac{\cos\alpha }{b_{2}} \end{array}$$ (34)

where *α* is the level inclination of anchors and *b*_2_ is the lateral distance between the adjacent anchors. In this case, λ shall be 0.47. Note that the calculation is assumed as a plane strain problem, the value of λ itself shall be reduced as well. Here we take the final reduction factor λ = 0.37 [12].

By trial and error, a parameter combination of *m* = 300 kN/m^4^, *n* = 1, and *m*_0_ = 0 showed the best fit to the measured data.

The design code *(JGJ, 2012)* suggests that the value of *m* should be determined through horizontal loading test and local experience, or using following Eq 35:

$$\begin{array}{c} m=\frac{0.2\varphi^{2}-\varphi +c}{v_{b}} \end{array}$$ (35)

where *c* and *φ* is cohesion and internal friction angle of soil, respectively. For multi-layer soil, values of *c* and *φ* shall take into consideration every layer of soil, and *v*_*b*_ is the displacement of retaining structure at pit bottom. While *v*_*b*_ ≤ 10*mm*, take *v*_*b*_ = 10*mm*.

In this case, the value of *m* when *n* = 1 and *m*_0_ = 0 is 1200 kN/m^4^ through field horizontal loading test, and it represents the overall properties of the soils. Some studies show that the value obtained through the test should be reduced by 65%-80% when used in plane problems [13]. Thus, in this case the reduction factor is 25% (= 300/1200).

**Fig 4:**
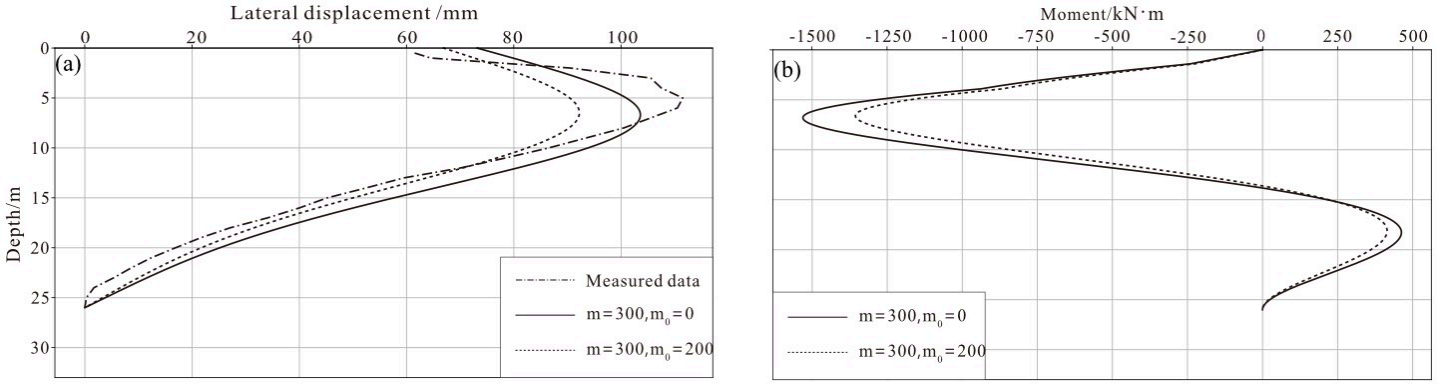
Relative displacement and moment of the retaining pile. a: Lateral displacement. b: Moment of the cross section.

The relative displacement and moment of the retaining pile are shown in Fig 4, and the absolute displacements of *m*_0_ = 0 and *m*_0_ = 200 at the lower end of the pile are 17.4 mm and 17.9 mm, respectively.

Note that the solution includes an infinite series (e.g., Eq 16), and a check was conducted to confirm its convergence. The displacements of the retaining pile at four typical depths were calculated when different numbers of terms in the series solution were taken. The results are shown in Table 2 and indicate that the solution has good convergence.

**Table 2:** Convergence check of series solution.

z	4 terms (mm)	6 terms (mm)	10 terms (mm)
0	73.12	73.16	73.16
1.9m	86.24	86.28	86.28
3.4m	94.27	94.31	94.31
6.8m	103.55	103.60	103.60

In addition, an analysis of the sensitivity of the parameters was performed. The displacement increment ratio of the retaining pile at four typical depths was calculated by assuming an error of 10% in the input parameter. Six cases were assumed: in case 1, it is assumed that the equivalent stiffness of the top beam was reduced by 10%; in case 2, the stiffness of the first anchor (at *z* = 1.4 m) was reduced by 10%; in case 3, the earth pressure was reduced by 10%; and in cases 4-6, the parameter value was increased by 10% corresponding to the reduced parameters in cases 1-3, respectively. All the cases are summarized in Table 3.

**Table 3:** Summary of six assumed cases.

	Case 1 (%)	Case 2 (%)	Case 3 (%)	Case 4 (%)	Case 5 (%)	Case 6 (%)
Stiffness of top beam	-10	-	-	10	-	-
Stiffness of first anchor	-	-10	-	-	10	-
Earth pressure	-	-	-10	-	-	10

The change percentage of displacement at each position in each case is shown in Table 4.

**Table 4:** Change percentage of displacement in six assumed cases.

z	Case 1 (%)	Case 2 (%)	Case 3 (%)	Case 4 (%)	Case 5 (%)	Case 6 (%)
0	4.45	3.55	-3.45	-4.08	-3.36	3.45
1.4m	3.67	2.81	-3.06	-3.09	-2.66	3.06
3.9m	2.00	1.81	-2.50	-1.83	-1.71	2.50
6.2m	1.22	1.96	-2.03	-1.11	-1.13	2.02

The results showed that the difference between the calculated results is only up to 4.45% with input data with a 10% error. In actual engineering practice, measurements of the mechanical properties of the field, such as earth pressure, stiffness of the top beam and stiffness of the anchor, are usually not very accurate and have an acceptable measurement error (*e.g*., 10%). The evaluation results in Table 4 indicate that this method can give stable calculation results based on the input parameters with acceptable measurement error. However, more case studies are necessary to give detailed standards on the adoption of parameters.

## Conclusion

This paper presents an analytical solution to calculate the displacements and internal forces of a pile-anchor retaining structure for excavation engineering and proposes a modified method to determine the most dangerous potential sliding plane based on the analytical solution and Sweden slice method. An example of the excavation was introduced to validate the solution. The main achievements are as described as follows:

1. The *singular function method* was introduced to solve the equilibrium differential equation of the retaining pile above the pit bottom through the direct integration method, while the equation of the lower part of the pile, which was a transcendental equation, was solved using the series method;
2. The solution was validated in an excavation project in Wenzhou, Zhejiang, China. The calculated displacement showed good agreement with the measured data. The convergence of the solution was also tested and the result shows that the solution has a good convergence. The reduction factor of m in this project is 25%. More case studies shall be taken to give detailed standards of parameters adoption.

## Supporting information

S1 Table Notations. Symbols used in the main text are summarized and listed in S1 Table.
(PDF)
