# Correction: Calculation of displacements and internal forces of anchored retaining piles

**DOI:** 10.1371/journal.pone.0307694

**Published:** 2024-07-18

**Authors:** Xiaoyi Yuan, Longzhu Chen, Jianliang Deng

In the Solution of differential equations subsection of Calculation of internal forces, there is an error in the Eqs 6 and 7. The multiplier *b*_1_ was missing in these two Equations. Please view the complete, correct equation here:

$$\begin{array}{c} EIy=b_{1}\overset{M}{\underset{i=1}{\sum }}\left[\frac{q_{i1}}{24}{\langle z-z_{i1}\rangle }^{4}-\frac{q_{i2}}{24}{\langle z-z_{i2}\rangle }^{4}\right.\left.+\frac{q_{i2}-q_{i1}}{120\left(z_{i2}-z_{i1}\right)}\left({\langle z-z_{i1}\rangle }^{5}-{\langle z-z_{i2}\rangle }^{5}\right)\right]\\ -\overset{N}{\underset{j=1}{\sum }}\frac{1}{6}k_{j}y_{j}{\langle z-h_{j}\rangle }^{3}+C_{3}z+C_{4} \end{array}$$ (6)

$$\begin{array}{c} EI\varphi =b_{1}\overset{M}{\underset{i=1}{\sum }}\left[\frac{q_{i1}}{6}{\langle z-z_{i1}\rangle }^{3}-\frac{q_{i2}}{6}{\langle z-z_{i2}\rangle }^{3}\right.\left.+\frac{q_{i2}-q_{i1}}{24\left(z_{i2}-z_{i1}\right)}\left({\langle z-z_{i1}\rangle }^{4}-{\langle z-z_{i2}\rangle }^{4}\right)\right]\\ -\overset{N}{\underset{j=1}{\sum }}\frac{1}{2}k_{j}y_{j}{\langle z-h_{j}\rangle }^{2}+C_{3} \end{array}$$ (7)
