# Supplementary material for: Calculation of displacements and internal forces of anchored retaining piles
Source: PLoS One. 2020 Dec 10;15(12):e0243659. doi: 10.1371/journal.pone.0243659 (PMC7728219; doi:10.1371/journal.pone.0243659)
Supplement: S1 Table — Symbols used in the main text are summarized and listed in S1 Table. (PDF) [file pone.0243659.s001.pdf]

S1 Table: Notations

M	Number of soil layers
N	Number of anchors
p_j	Resistance of the jth anchor acting on the retaining pile
k_j	Tensile stiffness of the jth anchor
y_j	Displacement of retaining pile at the position of jth anchor
q_i	Earth pressure of the ith soil layer
h_j	The distance of jth anchor from the top of pile
b_1	Calculative width of the pile
EI	Flexural rigidity of the retaining pile
K	Equivalent stiffness of soil below the pit bottom
p_h	Horizontal active pressure due to the soil layers above the pit bottom
m	Coefficient of *M-Method* and *Modified M-Method*
m_0	Coefficient of *Modified M-Method*
$\sum \gamma_i h_i$	Sum of the dead weight of the soil layers above the pit bottom
q_0	Ground overload
$\overline{c}$	Average cohesion weighted by the thickness of the soil layers above the pit bottom
$\overline{\varphi}$	Average internal friction angle weighted by the thickness of the soil layers above the pit bottom
C_3	Integral constant
C_4	Integral constant
φ_{b-}	Rotation calculated from the equilibrium differential equation above the pit bottom
y_{b-}	Displacement calculated from the equilibrium differential equation above the pit bottom
φ_{b+}	Rotation calculated from the equilibrium differential equation below the pit bottom
y_{b+}	Displacement calculated from the equilibrium differential equation below the pit bottom

φ_b	Rotation of the pile at the pit bottom
y_b	Displacement of the pile at the pit bottom
Q_b	Shear force of the retaining pile at the pit bottom
M_b	Bending moment of the retaining pile at the pit bottom
δ	Displacement of the lower end of the retaining pile
θ	Rotation of the lower end of the retaining pile
$K_{s,i}$	Safety factor of the ith slip arc
c_j	Cohesion of the jth soil slice at slip arc
φ_j	Friction angle of the jth soil slice at slip arc
d_j	Width of the soil slice
θ_j	Angle between the normal direction of the slip arc at the midpoint and the vertical direction
q_j	Attached load on the soil slice
ΔG	Dead weight of the soil slice
u_j	Water pressure
$R_k^{'}$	Uplift capacity of the anchors
α_k	Inclination of the kth anchor
θ_k	Angle between the normal direction of the slip arc at the intersection point of the anchor and the vertical direction
$s_{x,k}$	Lateral distance between the adjacent anchors
r_i	Radius of the slip arc
M_p	Calculated moment of the retaining pile at the intersection point of the slip arc and retaining pile
Q_p	Calculated shear force of the retaining pile at the intersection point of the slip arc and retaining pile
D_p	Vertical distance between the circle center of the slip arc and the intersection point of slip arc and retaining pile
c	Cohesion of soil
φ	Internal friction angle of soil

v_b	Displacement of retaining structure at pit bottom
λ	Reduction factor of stiffness of anchors when used in plane strain problem
α	Level inclination of anchors
b_2	Lateral distance between the adjacent anchors
